# Automated Identification of Common Disease-Specific Outcomes for Comparative Effectiveness Research Using ClinicalTrials.gov: Algorithm Development and Validation Study

**DOI:** 10.2196/18298

**Published:** 2021-02-08

**Authors:** Anas Elghafari, Joseph Finkelstein

**Affiliations:** 1 Center for Biomedical and Population Health Informatics Icahn School of Medicine at Mount Sinai New York, NY United States

**Keywords:** clinical trials, clinical outcomes, common data elements, data processing, ClinicalTrials.gov

## Abstract

**Background:**

Common disease-specific outcomes are vital for ensuring comparability of clinical trial data and enabling meta analyses and interstudy comparisons. Traditionally, the process of deciding which outcomes should be recommended as common for a particular disease relied on assembling and surveying panels of subject-matter experts. This is usually a time-consuming and laborious process.

**Objective:**

The objectives of this work were to develop and evaluate a generalized pipeline that can automatically identify common outcomes specific to any given disease by finding, downloading, and analyzing data of previous clinical trials relevant to that disease.

**Methods:**

An automated pipeline to interface with ClinicalTrials.gov’s application programming interface and download the relevant trials for the input condition was designed. The primary and secondary outcomes of those trials were parsed and grouped based on text similarity and ranked based on frequency. The quality and usefulness of the pipeline’s output were assessed by comparing the top outcomes identified by it for chronic obstructive pulmonary disease (COPD) to a list of 80 outcomes manually abstracted from the most frequently cited and comprehensive reviews delineating clinical outcomes for COPD.

**Results:**

The common disease-specific outcome pipeline successfully downloaded and processed 3876 studies related to COPD. Manual verification indicated that the pipeline was downloading and processing the same number of trials as were obtained from the self-service ClinicalTrials.gov portal. Evaluating the automatically identified outcomes against the manually abstracted ones showed that the pipeline achieved a recall of 92% and precision of 79%. The precision number indicated that the pipeline was identifying many outcomes that were not covered in the literature reviews. Assessment of those outcomes indicated that they are relevant to COPD and could be considered in future research.

**Conclusions:**

An automated evidence-based pipeline can identify common clinical trial outcomes of comparable breadth and quality as the outcomes identified in comprehensive literature reviews. Moreover, such an approach can highlight relevant outcomes for further consideration.

## Introduction

Over the past decade, comparative effectiveness research has taken center stage as a major vehicle to facilitate informed decisions on optimal treatment regimens as well as a means to improve health care at both the individual and population levels [1]. Comparative effectiveness research has been defined by the Institute of Medicine committee as “the generation and synthesis of evidence that compares the benefits and harms of alternative methods to prevent, diagnose, treat, and monitor a clinical condition or to improve the delivery of care. The purpose of comparative effectiveness research is to assist consumers, clinicians, purchasers, and policy makers to make informed decisions that will improve health care at both the individual and population levels” [2]. Randomized controlled trials (RCTs) are considered the gold standard for clinical effectiveness research [3].

RCTs are conducted to determine whether an intervention is effective by comparing outcomes between different arms of a study that are chosen to reflect beneficial and harmful effects [4]. Results of outcome comparison are used by decision makers to make evidence-based health care choices [5]. Thus, it is critical that study outcomes used in RCTs are relevant for the decision makers and allow cross-trial comparison especially when used to assess the same condition [6]. Recent studies demonstrated inconsistencies in choices of RCT outcomes, which limit potential cross-trial comparison and affect the reproducibility and overall usefulness of RCTs [7]. For example, a comprehensive review of oncology research found that more than 25,000 outcomes were reported only once or twice in oncology trials [8]. Differences in outcome definitions and measurements make it difficult or even impossible to synthesize results of different RCTs [9]. An analysis of missing data in systematic clinical trial reviews found that 71% of reviews could not obtain relevant key outcomes from the included trials [10]. Significant variation in outcome reporting has been noted by a recent systematic review of 109 RCTs assessing interventions for genitourinary symptoms associated with menopause [11].

Misalignment in clinical trial reporting could be addressed by the introduction of an agreed upon collection of common data elements (CDEs) [12]. The importance of developing CDEs for clinical trials, including common disease-specific trial outcomes, has been emphasized by researchers in various fields of medicine and public health [13-16]. Common trial outcomes can help researchers conduct cross-study aggregations and comparisons, facilitate meta-analyses, and increase reproducibility and efficiency. Sheehan et al [15] emphasized the importance of developing CDEs for clinical research generally and noted the current absence of “formal international specifications governing the construction or use of Common Data Elements.” Thurmond et al [13] discussed a multiagency scientific initiative to develop CDEs for traumatic brain injury and psychological health and noted that the “use of different measures to assess similar study variables and/or assess outcomes may limit important advances in (...) research. Without a set of common data elements (CDEs; to include variable definitions and recommended measures for the purpose of this discussion), comparison of findings across studies is challenging.”

With regard to clinical outcomes specifically (as the primary class of CDEs clinical trials are concerned with), a lot of emphasis has been placed on developing standardization approaches and addressing potential gaps. Ioannidis et al [17] examined the gaps in outcomes reported by clinical trials. In their survey of 174 systematic reviews with 1041 trials on preterm infants, they found that most trials were missing information on serious common outcomes for this population, and concluded by recommending the “use of standardized clinical outcomes that would have to be collected and reported by default in all trials in a given specialty.”

The traditional approach for the development of common outcomes for a particular field involves assembling panels of subject matter experts, who will then embark on an iterative multiphased deliberation process to identify the set of outcomes and agree on definitions and time frames. Redeker et al [16] offer a window into this, describing a process that involves “convening a working group, subdividing the working group based on areas of need, holding an introductory meeting, developing CDEs for assigned areas by subgroups, reviewing the work of all the subgroups, revising the CDEs based on feedback, obtaining public review of the identified CDEs, revising the CDEs based on feedback, and posting the first versions of the CDEs on the website.” Typically, this time-consuming and labor-intensive process does not employ automated or data-driven methods to systematically utilize information from ClinicalTrials.gov on the thousands of clinical trials relevant to the conditions under consideration.

ClinicalTrials.gov is the most comprehensive international clinical trial registry that contains over 350,000 research studies from 216 countries [18]. Registration with ClinicalTrials.gov includes submission of verified, detailed, and structured information pertinent to clinical trial design, study timeline, inclusion/exclusion criteria, primary and secondary outcomes, subject follow-up, and trial results. Data from ClinicalTrials.gov have found a variety of innovative uses in biomedical informatics research. For example, Huser and Cimino have worked to link ClinicalTrials.gov to PubMed to analyze the proportion of trials that reported results through publication [19] and to understand the quality and completeness of the links [20]. Anderson et al [21] used ClinicalTrials.gov data to study level of compliance with result reporting requirements. Bourgeois et al [22] used ClinicalTrials.gov data to compare industry-funded trials to nonindustry-funded trials in terms of the likelihood of reporting positive outcomes, while Hartung et al [23] investigated the discrepancies between results submitted to ClinicalTrials.gov’s results database and those published in peer-reviewed journals.

ClinicalTrials.gov data mining has been used to analyze the characteristics of oncology clinical trials [8], trends in clinical trials for stroke treatment [24], disparities in racial and ethnic representation in stem cell clinical trials [25], nonpublication rates of registered digital health trials [26], and relationships between mutations and phenotypes [27]. With regard to outcomes and other CDEs, Huser et al [19,20] examined the use of CDEs in real data sets and showed how the CDEs identified change by changing the threshold of commonness. Vodicka et al [28] analyzed the proportion and characteristics of ClinicalTrials.gov trials that included patient-reported outcomes. Luo et al [29] proposed a semiautomatic approach for identifying inclusion criteria CDEs. Mayer et al [30] collected variables from 15 HIV clinical trial dictionaries and clustered them using the Unified Medical Language System (UMLS). These efforts demonstrate the power of automated evidence-based approaches. However, the potential of ClinicalTrials.gov data to inform the development of clinical trial outcomes has received very little attention in the biomedical informatics literature.

Our aim in this work was to address the lack of automated evidence-based tools in the development of clinical outcomes by introducing our outcome identification pipeline and evaluating the technical correctness of its operations, as well as the quality and relevance of the clinical outcomes identified.

## Methods

### Data Source: ClinicalTrials.gov

The Food and Drug Administration Amendments Act of 2007 (FDAAA) (Section 801 of Public Law 110-85) requires an entity or individual who is responsible for an applicable clinical trial to submit the clinical trial information to the Clinical Trial Registry Data Bank (CTRDB). For the purposes of the FDAAA, ClinicalTrials.gov plays the role of the CTRDB maintained by the National Library of Medicine. ClinicalTrials.gov serves as a mandatory repository for clinical trials conducted under US regulatory oversight. Registration in ClinicalTrials.gov or a similar registry is a prerequisite for publishing clinical trial results in the majority of peer-reviewed journals.

The ClinicalTrials.gov portal supports self-service queries of registered clinical trials through a user interface at the website’s main page. The interface allows the user to search for a particular condition or disease by inputting its name into the “condition or disease” field or into the “other terms” field. In the former case, only trials that focus on the condition are returned. In the latter case, more results are returned, but they may not all be relevant to the condition. In parallel to the website, ClinicalTrials.gov offers a RESTful application programming interface (API) that allows automated submission of search queries (eg, from a computer program) and returns results in a computation-friendly format (eg, XML) for further processing.

**Figure 1:**
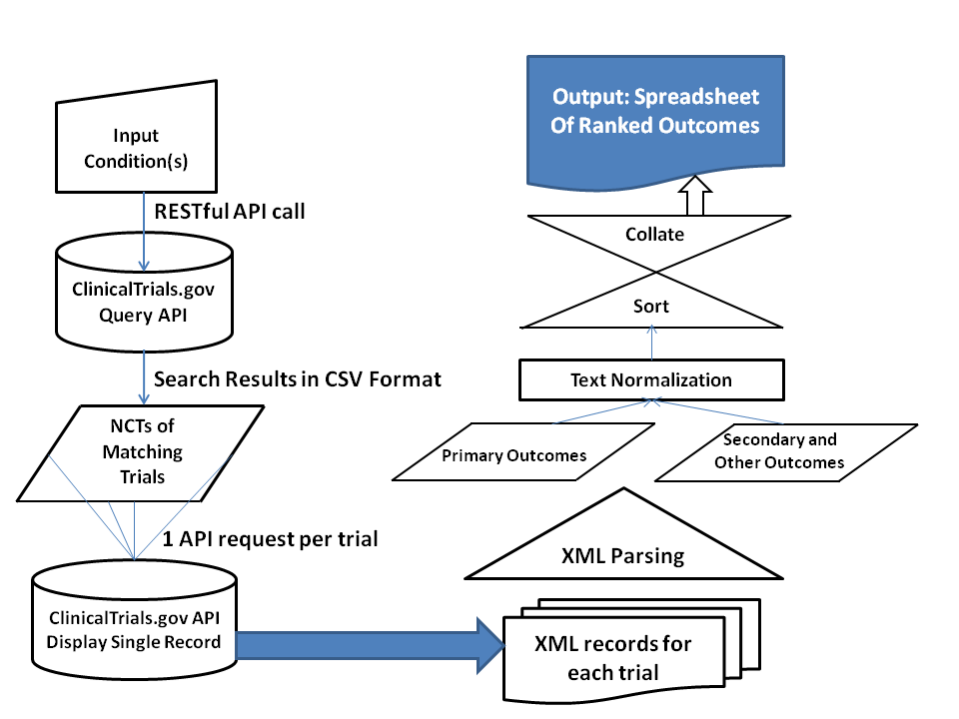
Pipeline workflow.

We implemented the ClinicalTrials.gov query pipeline using Python 3.7 [31] with libraries *URLLIB.request*, *Pandas*, and *Xml.etree*. In what follows, we provide a technical description of the components of the pipeline, representing the logical steps from the input query to the final output, and the list of collated and ranked clinical outcomes. A visual summary of the workflow is provided in Figure 1.

#### Step 1: Interfacing With the ClinicalTrials.gov API Search Endpoint

The input term, representing a condition such as “chronic obstructive pulmonary disease (COPD)”, is embedded into a URL that is used by the *URLLIB.request* module to interface ClinicalTrials.gov’s RESTful API at https://clinicaltrials.gov/ct2/results/download_fields?cond=COPD.

Other parameters of the API call include the following: the number of results to be returned per call (*down_chunk*), the results page (if the total number of matching results exceeds the number of results per page, the results will be broken into several chunks, and each must be accessed with a separate API call that references that page’s number), and the format of the results table (*down_fmt*, which can be specified as XML, CSV, PDF, etc).

This call mimics the search for the term using the “*condition or disease*” field on the user interface. By substituting “term” for “cond” in the URL, the call would return the same results obtained by querying for the term using the “*other terms*” field on the web page.

In our implementation, we set the number of *down_count* to 10,000 (the maximum that ClinicalTrials.gov’s API allows) and *down_chunk* to 1, which guarantees that most queries will return results contained in one chunk. For the few queries that yield over 10,000 matching trials (eg, “cardiovascular diseases” yields 39,704 results), the pipeline continues incrementing the *down_chunk* parameter and generating a new API call with the updated chunk number until the results are exhausted.

Each call returns a table of results in CSV format. Python’s Pandas library is used to parse the table into a Data Frame object. The output from this component is a list of Data Frames, with one for each chunk of each term’s results.

#### Step 2: Aggregating and Deduplicating the Results

The output of the interface with the search endpoint is a list of Data Frames (tables). Each table stores the details about the trials that match the input condition, and those details include the National Clinical Trial (NCT) number of each trial. The NCT number functions as a unique identifier for a registered study and can be used to download the full record of that study. In the case of multiple tables (due to multiple input terms or multiple pages of results returned by the API), it is necessary to aggregate the NCT numbers from all the tables and remove duplicate NCT numbers if they occur (this happens when the input terms are related, eg, “emphysema” and “COPD,” as many trials match both conditions). The output of this step is a list of unique NCT numbers that identify the trials matching the input conditions.

#### Step 3: Interfacing With ClinicalTrials.gov to Download the Trial Records

Having arrived at the list of NCT numbers for all the trials in the results, the next step involves interfacing with ClinicalTrials.gov again to download each result in XML. ClinicalTrials.gov allows obtaining a single record in XML by calling https://clinicaltrials.gov/ct2/show/NCT_Number?displayxml=true.

XML is a widely used markup format that most programming languages can work with and parse. Obtaining the trial data in XML sidesteps the challenges of parsing the exact text from a web page. Instead, the XML tree can be searched for the nodes with certain labels (eg, “primary_outcome” and “secondary_outcome”), and the values of those nodes are then immediately accessible in a structured manner. For each NCT number in our aggregated set of results, a URL call is made and the XML record of that study is saved for parsing in the next step.

#### Step 4: Parsing the XML for Primary, Secondary, and Other Outcomes and Time Frames

Having downloaded the XML records for the trials that match the input terms, the next step is to parse the clinical outcome names, descriptions, and time frames from the XML.

Our implementation uses Python’s built-in *xml.etree* module to parse the XML string into a tree. Then, the *iter()* function is called on the name of the nodes whose values will be extracted (*primary_outcome*, *secondary_outcome*, and *other_outcome*)*.* Each node has further children that record the name of the outcome (*measure*), description, and time frames. The pipeline parses out those elements and stores them along with the NCT number.

#### Step 5: Normalizing Outcome Texts and Building a Frequency Table

Following the parsing of outcomes in the previous step, the next step in the pipeline is to normalize those texts of those outcomes, group them, and rank them by frequency. The text normalization step is needed to handle the numerous heterogeneous ways for writing the same outcome name. Given an outcome string, the pipeline applies the following transformations:

1. If the outcome string ends with an abbreviation (letters between parentheses matching the initials of the words, eg, “Quality of life [QoL]”), remove the abbreviation.
2. Change the string to all lower case.
3. Replace all punctuation marks with a space.
4. Replace every occurrence of two or more consecutive space marks with one space mark, and strip the spaces from both ends of the string.

Then, the normalized form of the outcome text is stored in a hash table that maps each outcome string to the list of trials in which it is used. After all the outcome strings are normalized, the table is sorted by frequency of occurrences.

#### Step 6: Generating an Output Spreadsheet

In the final step, the pipeline uses the constructed frequency tables to generate a readable CSV spreadsheet of the clinical outcomes for the input condition. The spreadsheet consists of the following three columns: outcome name, number of trials in which it is used, and the NCT IDs of those trials (allowing the user to further explore trials).

### Evaluation Methods

Our evaluation of the pipeline consisted of the following two parts: (1) a technical evaluation that compares the pipeline’s output to the data accessible through ClinicalTrials.gov’s website to verify that the downloading and parsing steps are implemented correctly and (2) a systematic evaluation of the outcomes identified by the pipeline for COPD in comparison to the outcomes identified in published widely cited reviews.

#### Evaluating the Technical Correctness of the Download and Parsing Processes

We verified the technical correctness of the implementation by comparing the trials downloaded via the API to the trials that can be obtained from the ClinicalTrials.gov website’s self-service query interface. This involved verifying that for each query condition, the pipeline was downloading the number of trials as the number that appeared on the website when manually searching for that condition.

In addition, we verified that the XML parsing and collating were correct by sampling pairs of outcomes and NCT identifiers from the resulting spreadsheet, accessing the ClinicalTrials.gov webpages of those trials, and verifying that all the outcomes listed in the output are present on that page. We similarly evaluated the completeness of the pipeline’s output relative to the website by sampling in the other direction. Starting from the results obtainable from the website for a given condition, we sampled various trials and verified that each trial in the sample appeared in the output of the pipeline along with all the outcomes listed for it.

#### Evaluating the Quality of Pipeline-Identified Clinical Outcomes

To assess the quality and usefulness of the clinical outcomes that can be automatically identified by the pipeline, we selected COPD as a testing use case. COPD was chosen because of the availability of several frequently cited expert reviews delineating COPD-specific clinical outcomes for clinical research, which could serve as a gold standard for assessing the relevance of clinical outcomes generated by the automated pipeline from ClinicalTrials.gov data.

Clinical outcomes from four published systematic reviews [32-35] were manually abstracted by the authors, resulting in a total of 80 outcomes for COPD clinical trials. These four reviews represent the most widely cited publications systematically analyzing outcome measures in COPD trials during the last 15 years. These reviews were conducted manually by internationally recognized expert teams, and they were based on overall 389 articles referenced in these publications. The automated pipeline used four query terms related to the condition (*COPD*, *chronic obstructive pulmonary disease*, *emphysema*, and *chronic bronchitis*) to generate pipeline-identified outcomes that were compared to the outcomes manually abstracted from the expert reviews.

#### Evaluation Metrics

The quality of the automated ClinicalTrials.gov pipeline for clinical outcome generation was assessed using recall and precision. Treating the literature review outcomes as the gold standard, every pipeline-identified outcome that appeared in the gold-standard set was a “true positive” (TP) prediction, every pipeline-identified outcome not appearing in the gold standard set was a “false positive” (FP) prediction, and every outcome from the literature reviews not identified by the pipeline was a “false negative” (FN) prediction for the pipeline. Recall was considered the ratio of TP to (TP + FN), while precision was the ratio of TP to (TP + FP). Intuitively, recall measures the coverage of our pipeline relative to the benchmark, and a low recall would mean the pipeline is failing to identify many benchmark outcomes. Precision, on the other hand, measures how many of the pipeline outcomes are the same as the benchmark outcomes, and a low precision indicates that the pipeline is identifying many outcomes that do not appear in the benchmark set.

## Results

### Correctness of the Data Downloading and Parsing

In evaluating the technical correctness of the output, we employed a number of testing conditions and terms, and compared the result count from both the pipeline and the website. There was a perfect match between the two in all cases, indicating no loss of data that the pipeline is obtaining from the API as compared to the website. Our evaluation of samples of the outcomes and trials similarly indicated a perfect match between the data obtained from the website and the output of the pipeline, with the only difference being the intentional normalization by the pipeline of the outcome texts described in step 5 of the pipeline operation.

Table 1 provides general statistics related to our application of the pipeline for COPD-related terms. The number of trials collected for each term was the same as can be seen on the ClinicalTrials.gov website on January 22, 2020 (number of trials for a given condition can increase over time as new studies are registered). As can be seen in Table 1, the number of trials generated by querying the “other terms” field was higher than that generated by querying the “condition” field, as the former includes a search of additional fields.

**Table 1:** General statistics.

Variable	Querying using the “condition” field	Querying using the “other terms” field
Number of trials downloaded using the query term “COPD”	3596	4201
Number of trials downloaded using the query term “chronic obstructive pulmonary disease”	2993	3341
Number of trials downloaded using the query term “emphysema”	414	592
Number of trials downloaded using the query term “chronic bronchitis”	182	244
Number of unique trials (after removing duplicates)	3876	4450
Number of unique trials with outcomes listed	3734	4299
Percentage of trials listing outcomes	96.3%	96.6%
Number of primary outcomes parsed	5856	7033
Number of secondary/other outcomes parsed	16,016	18,872
Time required by the pipeline to download and parse the trials	10 minutes 34 seconds	13 minutes 35 seconds

#### Comparing the Automatically Identified Clinical Outcomes to Published Reviews

On comparing the outcomes identified automatically by the pipeline to the 80 outcomes abstracted from four widely cited reviews [32-35], we found matches for 74 of the 80 manually abstracted ones, giving the pipeline an overall recall of 92%. Tables 2 and 3 list the top primary and secondary pipeline outcomes, while Table 4 lists the four reviews’ outcomes that appeared in more than two reviews. Multimedia Appendix 1 shows the full mapping of the reviews’ outcomes to the automatically identified ones.

**Table 2:** Top 15 primary outcomes identified by the pipeline for chronic obstructive pulmonary disease.

Primary outcome	Occurrences as primary, n
Mortality	32
FEV1^a^	30
Quality of life	25
Forced vital capacity	14
Exercise capacity	14
Adverse events	13
Dyspnea	10
Lung function	10
COPD^b^ assessment test	9
Endurance time	9
Functional capacity	9
Safety	9
Oxygen saturation	8
Six-minute walk test	7
Maximum plasma concentration	7

^a^FEV1: forced expiratory volume in 1 second.

^b^COPD: chronic obstructive pulmonary disease.

**Table 3:** Top 15 secondary outcomes identified by the pipeline, excluding outcomes occurring frequently as primary.

Secondary outcomes	Occurrences as secondary, n
Heart rate	30
Length of hospital stay	25
St. George’s Respiratory Questionnaire	24
Blood pressure	21
Physical activity	20
Inspiratory capacity	18
Body composition	18
Time to first COPD^a^ exacerbation	16
Physician’s global evaluation	16
Depression	15
Body mass index	15
Hospital anxiety and depression scale	13
Patient satisfaction	13
Use of rescue medication	9
Cost-effectiveness	9

^a^COPD: chronic obstructive pulmonary disease.

**Table 4:** Top outcomes abstracted from published reviews.

Outcome^a^	Source (references)
Baseline Dyspnea Index	[32-35]
Transition Dyspnea Index	[32-35]
Borg Dyspnea Scale	[32-35]
Medical Research Council Dyspnea Scale	[32-35]
Chronic Respiratory Disease Questionnaire	[32-35]
St. George’s Respiratory Questionnaire	[32-35]
Body mass index, airflow obstruction, dyspnea, and exercise capacity	[32-34]
Six-minute walk test	[32-35]
Incremental shuttle walk test	[32-35]
SpO_2_: peripheral oxygen saturation	[32-34]
Forced expiratory volume in 1 second (FEV1)	[32-35]
Forced vital capacity (FVC)	[32-35]
FEV1/FVC	[32-35]
Static lung volumes	[33-35]
Number of exacerbations	[33-35]
Mortality	[33-35]

^a^Outcomes that appear in three or more reviews are shown. The full list of 80 outcomes and their equivalent from the pipeline can be seen in Multimedia Appendix 1.

While calculating the pipeline recall of the pipeline’s output, we searched for the 80 outcomes abstracted from the expert reviews and found 74 of them among the automatically generated outcomes, thus yielding recall of 92%. For calculating the pipeline precision as described in the methods section, the entire pipeline output required manual review of all automatically generated outcomes since many of them represented the same concept but were phrased differently and used a different abbreviation or spelling. To streamline this part of the assessment, only outcomes used in four or more clinical trials were considered for grouping, which eventually yielded a total of 96 pipeline outcomes. We evaluated each of those to see if they had an equivalent among the 80 outcomes abstracted from the expert reviews. Overall, 76 of the grouped outcomes had equivalent counterparts among the outcomes abstracted from the expert reviews, yielding a precision of 79%.

#### Examining the Differences Between Pipeline Outcomes and Review Outcomes

To better understand the quality of the pipeline’s output, we looked at the difference in results between what the pipeline generated and the outcomes from the literature. Textbox 1 lists the review outcomes that had no equivalent in the pipeline output, while Table 5 lists the top pipeline outcomes that had no equivalent in the abstracted reviews.

**Top false negatives (outcomes from the abstracted reviews with no match among pipeline-identified outcomes).**

**Outcomes**

Nottingham Health Profile

Medical Outcomes Study 6-Item General Health Survey (MOS-6A)

Symptom Severity Index

Two-minute step-in-place test

Time spent in weight-bearing activities

Sputum visual analog scale

Manchester Respiratory Activities of Daily Living Questionnaire

**Table 5:** Top false positives (outcomes generated by the pipeline but not appearing in any of the abstracted reviews).

Outcome	Frequency
Sleep quality	14
Self-efficacy	10
Pharmacokinetics	10
Berg balance scale	9
Maximum plasma concentration	9
Duration of mechanical ventilation	7
Pulmonary vascular resistance	7
Diaphragmatic function	6
Cognitive function	6
Timed up and go	5
Patient activation	5
Neural respiratory drive	5
Handgrip strength	4
Short physical performance battery	4
Severe Respiratory Insufficiency Questionnaire	4

## Discussion

### Principal Findings

We have introduced a general automated pipeline for evidence-based generation of clinical outcomes using data from ClinicalTrials.gov. We evaluated the quality of the generated outcomes for COPD by comparing to a list of outcomes collected from four comprehensive reviews. We found great overlap between the autoidentified outcomes and the manually abstracted ones. Treating the review outcomes as the gold standard, the pipeline results achieve 0.92 recall overall and 0.79 precision for the top outcomes (used in more than three studies).

In investigating the cause for lower precision relative to recall, we examined the FPs (those outcomes that are identified by the pipeline but are not part of the benchmark set). Table 5 lists the most frequent pipeline FPs. We find that most of these FPs appear relevant to the underlying condition (COPD) yet have not been covered in any of the four reviews we considered. This argues that the relatively low precision is not due to the pipeline generating irrelevant outcomes, but rather the pipeline identifying outcomes not included in the benchmark set. This points to the potential of this automated evidence-based approach to highlight measures and domains that might be underused in the literature.

### Limitations

While the results are encouraging, there are two main limitations to the data-driven evidence-based approach. First, there is a great deal of fragmentation in how the same outcome could be described when the data are entered into the trial registry, which leads to a large number of overlapping outcomes being identified. While the text normalization module can handle surface-level variations, some of the variations will require specialized ontologies (eg, to recognize that “spirometry” and “FEV1” are related outcomes). Some variations will still require some human judgment (eg, should “number of readmissions” and “number of hospitalizations” be grouped together for more compactness of the results or is the semantic difference sufficient to warrant keeping them distinct?).

The second limitation is that while the pipeline can be very useful in giving a data-backed view of the most frequent outcomes, it cannot replace the traditional role of experts in providing guidelines for which outcomes are suitable to use in a given situation. Combining the proposed evidence-based pipeline with expert analysis has the potential to greatly facilitate traditional workflow for CDE development. Recent publication evaluating methodology for the development of clinical outcome sets expressed concerns that the currently accepted methodology relies entirely on agreement and lacks alternatives [36,37]. Methods used in the selection of instruments for outcomes included in trial outcome sets can be improved by including automated means for identifying common disease-specific outcomes used in registered clinical trials [38,39].

There are many studies that used ClinicalTrials.gov data for systematic analysis. However, most of those studies focused on analyzing the quality and compliance of the data on ClinicalTrials.gov. For example, Huser and Cimino linked records of interventional studies to PubMed publications and showed that a large segment of trial sponsors failed to meet their mandate in publishing trial results [20]. Compliance with result reporting obligations was also the focus of the work by Anderson et al [21]. Other studies have also utilized ClinicalTrials.gov data to cluster clinical trials with similar eligibility criteria features [40], to characterize semantic heterogeneity of data elements [41], and to analyze nonpublication rates of registered clinical trials [26].

With respect to CDEs, there have been very few efforts to make use of ClinicalTrials.gov’s data. Kentgen et al [42] collected data from patient care forms related to acute coronary syndrome and then used the UMLS to semantically annotate and generate a list of the most common data elements. As in our study, the authors noted a lack of standardized and semantically enriched documentation for clinical outcomes. In another study, Holz et al [43] used UMLS to identify and harmonize a semantic core of CDEs for acute myeloid leukemia. However, neither of these studies used data from ClinicalTrials.gov. Among the few works that made use of trial registry data for CDE identification, Luo et al [29] proposed a semiautomatic approach for identifying disease-specific eligibility criteria. They used UMLS semantic types to parse CDEs from inclusion criteria free text. Their results showed that an automated approach can achieve very good performance compared with human annotators. The main difference between their work and ours is that they focused on eligibility criteria CDEs while we focused on clinical outcomes.

Vodicka et al [28] analyzed the proportion of trials that used patient-reported outcomes. While their work similarly includes parsing of ClinicalTrials.gov data, the focus of their analysis was characterizing the temporal trends of the usage of a predefined class of outcomes and the variation by sponsor type. To the best of our knowledge, this is the first report that focuses on the automated identification of clinical outcomes and evaluates the coverage of the identified outcomes by comparing to comprehensive and widely cited reviews.

### Future Directions

For future work, we plan to address the fragmentation issue by using the UMLS [44] in conjunction with the MetaMap API [45] and ontologies on BioPortal [46] to cluster related outcomes and allow the user to explore them by outcome domain or measure. According to Huser et al [47], optimal analyses of CDEs require engagement of multiple data sources and biomedical ontologies as well as real-world research use cases.

Furthermore, we believe that there is a lot of potential in the other data elements that ClinicalTrials.gov provides. This includes time frames of the outcomes as well as the Medical Subject Heading (MeSH) terms. For the time frames, the pipeline is currently parsing them along with the outlines, and the next step would be to fine tune the parsing and aggregation of time frames to include them in the output of the pipeline. MeSH terms are potentially very useful in aiding the classification and navigation of the variety of extracted outcomes. Since those MeSH terms typically include information about additional conditions, inclusion criteria, and intervention types, grouping the outcomes by the associated MeSH terms can offer the user of the data a way to zoom in and zoom out as needed.

### Conclusions

ClinicalTrials.gov offers a wealth of data that has not been fully utilized. An automated pipeline that leverages these data to identify relevant clinical outcomes for any given condition can greatly aid the traditional processes around clinical outcome selection and facilitate clinical trial fidelity and comparability.

## Appendix

Multimedia Appendix 1 Outcomes abstracted from the literature reviews.

## Abbreviations

API: application programming interface
CDE: common data element
COPD: chronic obstructive pulmonary disease
CTRDB: Clinical Trial Registry Data Bank
FDAAA: Food and Drug Administration Amendments Act
FN: false negative
FP: false positive
MeSH: Medical Subject Headings
NCT: National Clinical Trial
RCT: randomized controlled trial
TP: true positive
UMLS: Unified Medical Language System
