# Supplementary material for: Automated Identification of Common Disease-Specific Outcomes for Comparative Effectiveness Research Using ClinicalTrials.gov: Algorithm Development and Validation Study
Source: JMIR Med Inform. 2021 Feb 8;9(2):e18298. doi: 10.2196/18298 (PMC7899806; doi:10.2196/18298)
Supplement: Multimedia Appendix 1 [file medinform_v9i2e18298_app1.docx]

### Appendix 1

The following table shows all 80 clinical outcomes used as a benchmark, the reviews they were abstracted from, and an example of a registered trial the pipeline used to identify the outcome (n/a - indicates an outcome the pipeline did not identify)

| **Outcome from Literature** | **Sources** | **A Trial Used by the Pipeline to Identify this Outcome** |
| --- | --- | --- |
| BDI: Baseline Dyspnea Index | [15][16][17][48] | NCT01796730 |
| TDI: Transition Dyspnea Index | [15][16][17][48] | NCT01972776 |
| Dyspnea Visual Analog Scale | [15] | NCT03615040 |
| Borg Dyspnea Scale | [15][16][17][48] | NCT02567474 |
| Modified Borg Dyspnea Scale | [15] | NCT00274521 |
| MRC: Medical Research Council Dyspnea Scale | [15][16][17][48] | NCT00884962 |
| mMRC: modified Medical Research Council Dyspnea Scale | [15] | NCT00391612 |
| NYHA: New York Heart Association Functional Class | [15] | NCT01758744 |
| ADL: Activity of Daily Living | [15][17] | NCT00135538 |
| CRQ: Chronic Respiratory Disease Questionnaire | [15][16][17][48] | NCT00542932 |
| SGRQ: St George Respiratory Questionnaire | [15][16][17][48] | NCT02152605 |
| SF-36 Short Form (36-Item) Health Survey | [15][17] | NCT03981783 |
| EQ-5D: EuroQol 5D | [15] | NCT04146948 |
| CAT: COPD Assessment Test | [15][17] | NCT01204034 |
| FACIT: Functional Assessment of Chronic Illness Therapy | [15] | NCT01650480 |
| CCQ: Clinical COPD Questionnaire | [15] | NCT00690482 |
| Feeling thermometer | [15] | NCT02441725 |
| NHP: Nottingham Health Profile | [15] | n/a |
| MYMOP: Measure Yourself Medical Outcome Profile | [15] | NCT02330952 |
| BPQ: Breathing Problems Questionnaire | [15] | NCT00159549 |
| MOS-6A: Medical Outcomes Study 6-Item General Health Survey | [15] | n/a |
| GOLD: Global Initiative for Chronic Obstructive Lung Disease stage | [15][48] | NCT02041000 |
| EXACT-PRO: Exacerbations of Chronic Pulmonary Disease Tool | [15][48] | NCT02138916 |
| SSI: Symptom Severity Index | [15] | n/a |
| HADS: Hospital Anxiety and Depression Scale | [15] | NCT00792974 |
| Sputum Visual Analog Scale | [15] | n/a |
| CASA-Q: Cough and Sputum Assessment Questionnaire | [15] | NCT03107494 |
| Breathlessness, Cough, and Sputum Scale | [15] | NCT02250027 |
| London Chest Activities of Daily Living Scale | [15] | NCT03251781 |
| Barthel Index | [15] | NCT03111004 |
| Manchester Respiratory Activities of Daily Living Questionnaire | [15] | n/a |
| BODE: body mass index, airflow obstruction, dyspnea, and exercise capacity | [15][16][17] | NCT00250679 |
| 6-Min Walk Test | [15][16][17][48] | NCT00629239 |
| ISWT: Incremental Shuttle Walk Test | [15][16][17][48] | NCT00542932 |
| ESWT: Endurance Shuttle Walk Test | [15][17] | NCT00925171 |
| 3-Min Step Test | [15] | NCT01655199 |
| 3-Min Walk Test | [15] | NCT00807534 |
| 2-Min Step-in-Place Test | [15] | n/a |
| SpO2: Peripheral Oxygen Saturation | [15][16][17] | NCT03282019 |
| FEV1: Forced Expiratory Volume in 1 second | [15][16][17][48] | NCT00205920 |
| FVC: Forced Vital Capacity | [15][16][17][48] | NCT00152984 |
| FEV1/FVC | [15][16][17][48] | NCT02122627 |
| PEF: Peak Expiratory Flow | [15][48] | NCT00152984 |
| CRS: Computerized Respiratory Sounds | [15] | NCT02050711 |
| Fat-free Mass Index | [15] | NCT03438019 |
| BMI: Body Mass Index | [15] | NCT01786720 |
| Accelerometry | [15][17] | NCT02770417 |
| Time Spent in Weight-Bearing Activities | [15] | n/a |
| Maximal Voluntary Isometric Contraction | [15] | NCT02283580 |
| Quadriceps Twitch Responses | [15] | NCT00159367 |
| Maximum Inspiratory Pressure | [15] | NCT01945398 |
| Compliance | [16][48] | NCT00581087 |
| Static Lung Volumes | [16][17][48] | NCT00530842 |
| Inspiratory Capacity | [17][48] | NCT00123422 |
| Airway Resistance | [16][48] | NCT00677560 |
| Specific Airway Conductance | [48] | NCT03673670 |
| Diffusion Capacity | [16] | NCT00792974 |
| Arterial Blood Gases | [16][48] | NCT00994552 |
| Time to First Exacerbation | [16][17] | NCT00144339 |
| Number of Exacerbations | [16][17][48] | NCT00240435 |
| Severity and Duration of Exacerbations | [17][48] | NCT00788645 |
| Emergency Department Visits | [16][48] | NCT02944591 |
| Number of Hospitalizations | [16][48] | NCT00702078 |
| Number of ICU Admissions | [16][48] | NCT03028805 |
| Mortality | [16][17][48] | NCT00132860 |
| Lung Density by Computed Tomography (CT) Scan | [16][48] | NCT02719184 |
| CRP: C-reactive protein | [48] | NCT02740686 |
| Interleukin [IL]-8 | [16][48] | NCT00147017 |
| CD-8 Lymphocytes | [16][48] | NCT01509677 |
| Bronchoalveolar Lavage | [16][48] | NCT02272634 |
| Exhaled Breath Condensates | [16][48] | NCT01970618 |
| Circulating Inflammatory Markers | [16][48] | NCT01151306 |
| Use of Rescue Medications | [16][48] | NCT00062582 |
| Physician’s Global Evaluation | [16][48] | NCT00122434 |
| Subject’s Global Evaluation | [16][48] | NCT00359788 |
| Dynamic Hyperinflation | [17] | NCT01273298 |
| Static Hyperinflation | [17] | NCT02884830 |
| TLC: Total Lung Capacity | [17] | NCT01204034 |
| FRC: Functional Residual Capacity | [17][48] | NCT00500318 |
| RV: Residual Volume | [17] | NCT00391612 |
